# First-time use of a porcine small intestine submucosal plug device to close an acquired tracheo-esophageal fistula

**DOI:** 10.1186/s40792-023-01670-3

**Published:** 2023-06-09

**Authors:** Laura Dessard, Jacques Deflandre, Justine Deflandre, Vincent Moonen, Nicolas Delhougne, Yves Goffart

**Affiliations:** 1 Department of Otorhinolaryngology, Citadelle Hospital, Liège, Belgium; 2 Department of Gastro-Enterology, Citadelle Hospital, Liège, Belgium

**Keywords:** Tracheoesophageal, Fistula, Acquired, Small intestine submucosal plug device, Endoscopic technique

## Abstract

**Background:**

Acquired tracheo-esophageal fistula (TEF) is a rare, life-threatening pathology, responsible for severe comorbidities. Its management is a real therapeutic challenge and remains controversial.

**Case presentation:**

We report the first case of endoscopic treatment of TEF by using a porcine small intestine submucosal (SIS) plug device in a young quadriplegic patient after failed surgical closure by cervicotomy. After 1 year of follow-up, oral feeding of the patient was resumed and no clinical signs of fistula recurrence were evident.

**Conclusion:**

To our knowledge, we obtained for the first time, a satisfactory result for TEF closure with the use of a porcine SIS plug.

## Background

Tracheo-esophageal fistula (TEF) can be congenital or, more rarely, acquired. Prolonged mechanical ventilation by orotracheal intubation is the most common cause of benign, acquired TEF (75% of cases) [1–3]. TEF leads to numerous pulmonary complications and malnutrition. Multiple therapeutic strategies ranging from endoscopic approaches to cervicothoracotomy are available for the management of TEF. However, recurrence and postoperative complications occur frequently. Currently, there is no gold standard for management of this condition. Thus, therapeutic choices are complex and debatable. In the last few years, new endoscopic closure techniques have emerged to reduce the morbidity and mortality associated with cervicothoracic surgery. Herein, we report the first case on the endoscopic management and closure of a tracheo-esophageal fistula using a porcine small intestine submucosal (SIS) plug in a 27-year-old patient after the failure of cervicotomy.

## Case presentation

**Fig. 1:**
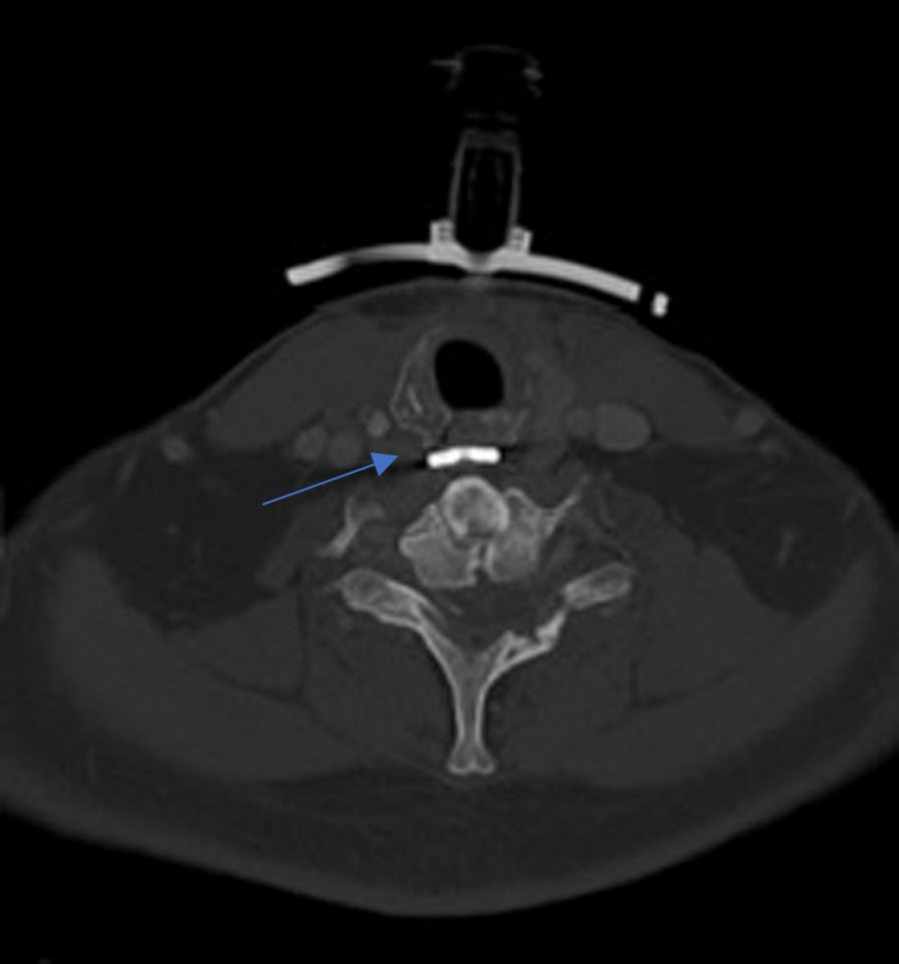
Cervical CT. Necrosis of the lamina of the cricoid cartilage, next to the osteosynthesis material (blue arrow)

**Fig. 2:**
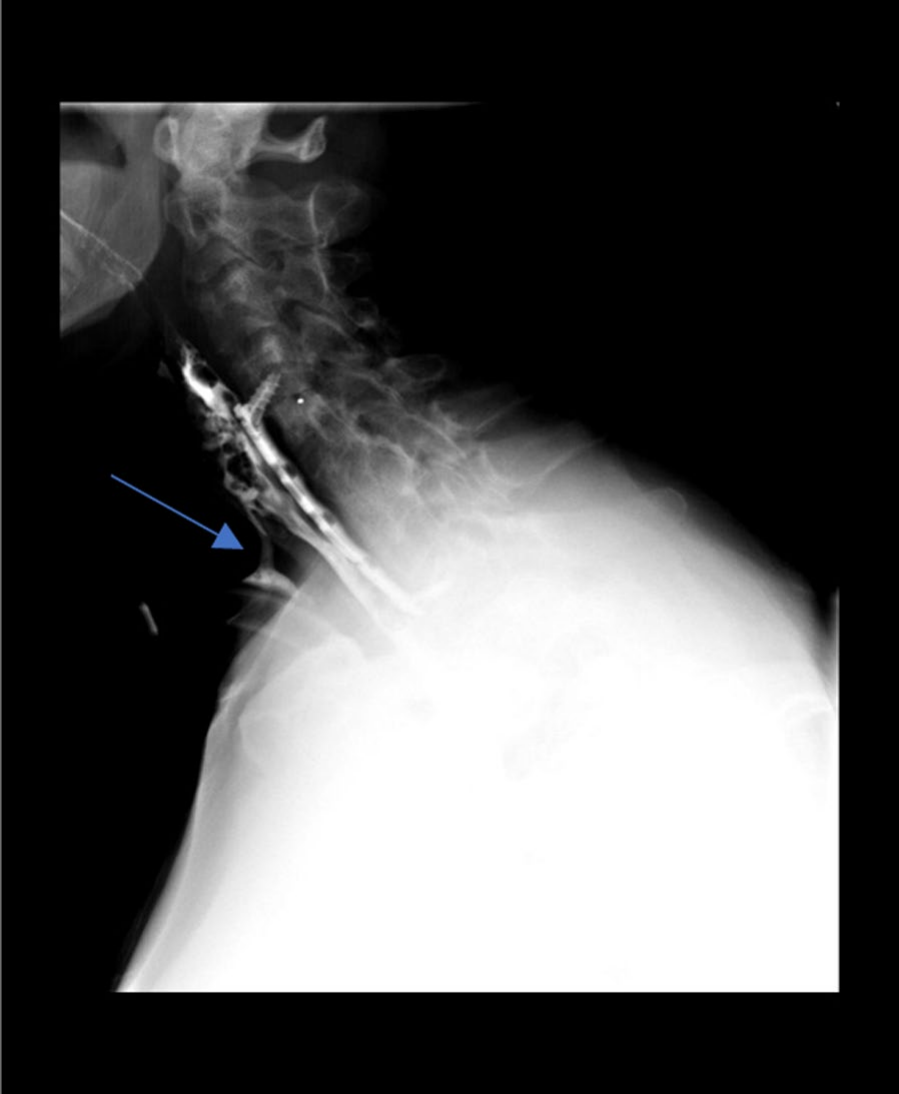
Contrast study. Extravasation of contrast material into the tracheal lumen (blue arrow)

**Fig. 3:**
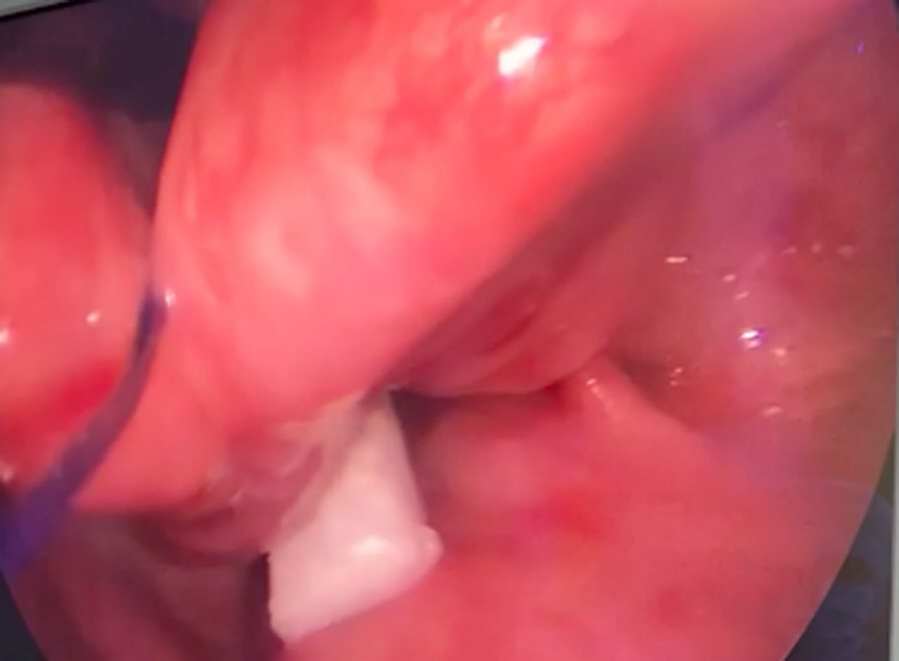
The plug is pulled by a wire guide through the retro-cricoid region

**Fig. 4:**
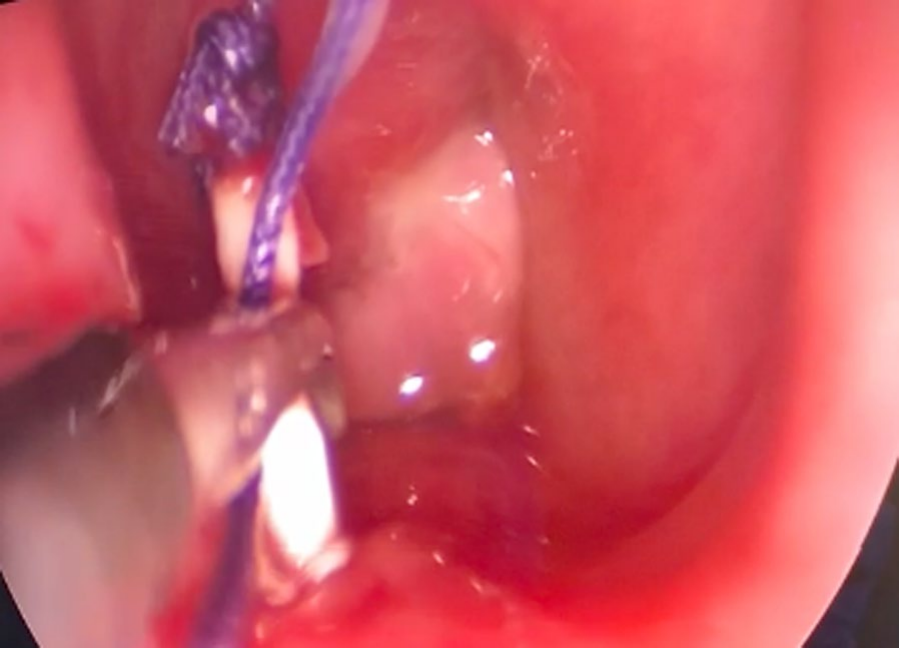
The plug in the tracheal lumen, once passed through the fistula orifice

**Fig. 5:**
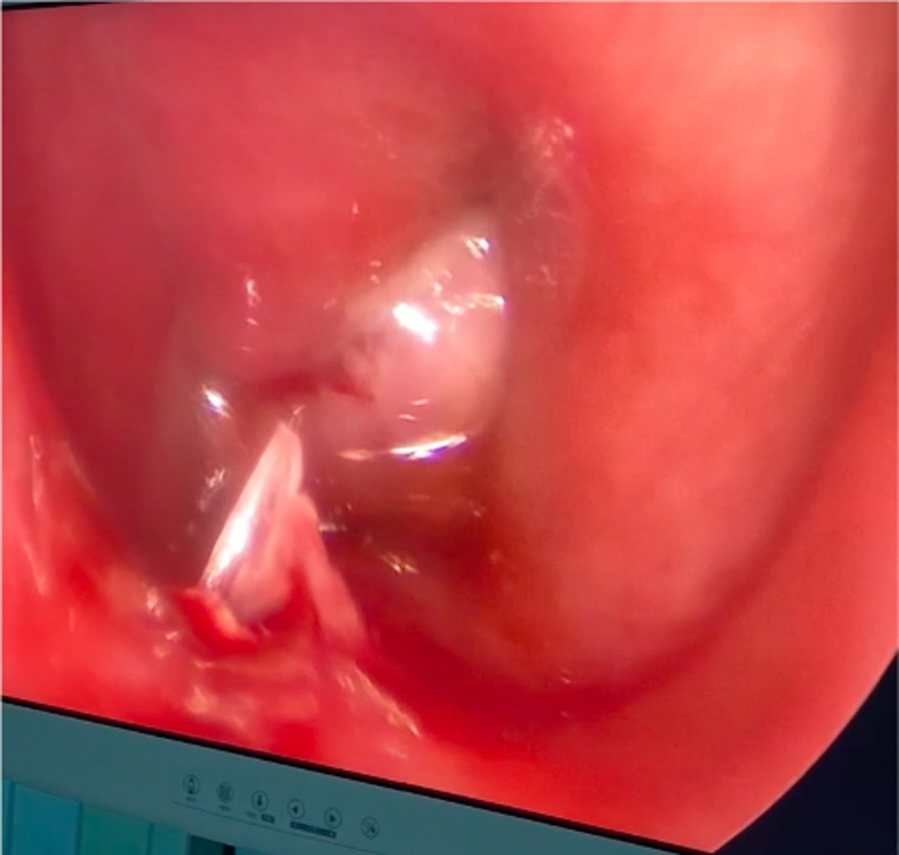
Final position of the plug in the tracheal lumen

**Fig. 6:**
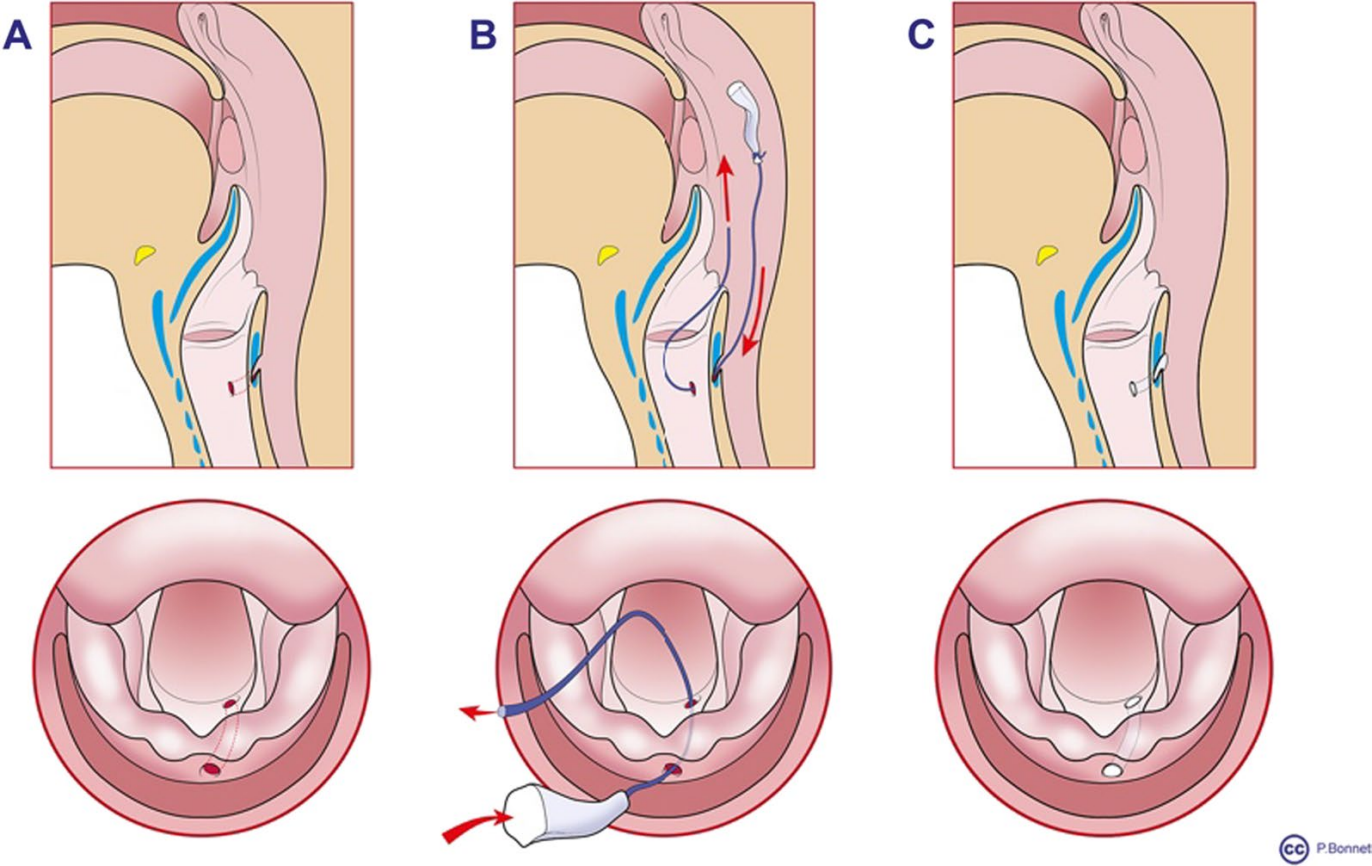
Surgical procedure illustrating in 3 steps. **A** The fistula path. **B** Insertion of the plug attached to the wire guide. **C** Closure of the fistula after plug insertion

A 27-year-old man was referred to our otolaryngology department for the management of a TEF that occurred after a road traffic accident, requiring a long stay in the intensive care unit with prolonged mechanical ventilation by orotracheal intubation, which was followed by tracheotomy. The patient had sustained multiple cervical fractures that required cervical osteosynthesis (from the 4th cervical vertebra to the 1st dorsal vertebra). The combination of prolonged intubation, with the presence of the cervical osteosynthesis material and the use of a nasogastric feeding tube, led to chondritis and progressive necrosis of the lamina of the cricoid cartilage, as shown on the cervical CT scan (Fig. 1). Barium swallow contrast confirmed the presence of tracheo-esophageal communication (Fig. 2). The patient underwent cervical surgery with strap muscle flap interposition in another institution. However, early recurrence of coughing after swallowing was observed, suggesting persistence of the fistula. When the patient arrived at our department, he was dependent on gastrostomy tube and tracheotomy for chronic aspiration. We performed rigid laryngoscopy and esophagoscopy, both of which confirmed persistence of the fistula. The fistula was located in the right retro-cricoid region, just above the esophagus, and traveled medially to the subglottic region, approximately 3 cm below the vocal cords. In association with the gastroenterology team, we proposed the use of a plug made of porcine SIS, a natural extracellular matrix made of collagen and other non-collagenous components (like fibronectin, glycosaminoglycans, proteoglycans, growth factors), that will constitute a natural guide to cell growth and ultimately to tissue healing. This plug is usually used in the management of anorectal and rectovaginal fistula. We performed the surgery, under general anesthesia using an endoscopic approach. After a good exposure of the fistula was achieved, the plug was introduced from the retro-cricoid region into the fistulous path with the help of a guide wire (Fig. 3), which was directed toward the trachea (Fig. 4). The excess plug flowing into the tracheal lumen was resected (Fig. 5). No fixation of the plug was necessary, which appeared stable, locked in the fistula path due to its flexibility and conical shape. The procedure is illustrated in Fig. 6. No intraoperative complications occurred. During the postoperative period, the patient was allowed to eat 1 week after the procedure. At 1 year of follow-up, there was no evidence of fistula recurrence. The patient could eat without any difficulty, and there was no sign of pulmonary infection.

## Discussion

TEF is a life-threatening condition, and its management remains debatable. Currently, there are no established recommendations for the management of TEFs because of the small number of randomized controlled studies. Surgical intervention is often unavoidable because of the low rate of spontaneous closure [1, 2]. Different types of procedures range from minimally invasive endoscopy to open cervicothoracic surgery. However, the surgical approach depends on the location of the fistula, its size, length, distance from the vocal cords, and tracheal anatomy (stenosis, malacia) [1, 2]. It is not uncommon to see tracheal stenosis coexisting with TEF [1, 2]. Therefore, accurate preoperatively identification of the fistula is essential to optimize the therapeutic result [3]. Bronchoscopy and esophagoscopy, rigid or flexible, and barium swallow if the patient is able to perform it, are the standard diagnostic methods [1, 3].

Many surgical techniques have been described, such as direct suture of the trachea and esophagus with or without interposition of a muscle flap, fascia, or resorbable patch [2, 4, 5]. The interposition of a muscle flap is used in most cases to ensure tissue vascularization, especially when the fistula is caused by prolonged mechanical ventilation with ischemic damage [2, 4, 5]. Tracheal resection with reconstruction is mostly reserved for large, circumferential TEF or those associated with tracheal stenosis [2, 4]. Some large, unresectable fistulas can be bypassed using a silicone tracheal T-tube [4]. Fistulas in laryngectomized and/or esophagectomized patients are the most difficult to treat and have the highest rate of recurrence [4]. In some cases, sternotomy and/or thoracotomy may be necessary to remove the fistula [3]. The advantage of these open surgical techniques lies in good exposure of the fistula; however, these techniques are associated with higher morbidity. Even if some authors report satisfactory long-term results (up to 94% fistula closure), these major surgeries have a morbidity varying between 15 and 56% and can lead to numerous postoperative complications in patients, often with poor general condition [1–5]. Tracheal stenosis, dehiscence, granuloma at the suture sites, or damage to the recurrent laryngeal nerves can be observed [1, 2, 5]. Fistula recurrence is also common (in up to 15% of cases) [2, 4, 6]. The period before the patient could resume oral feeding was also long. Therefore, it is essential to prepare the patient preoperatively with a gastrostomy tube renutrition, if necessary [2, 3]. Weaning the patient from mechanical ventilation preoperatively is also recommended [3, 4], when feasible, as well as appropriate antibiotic therapy in cases of pulmonary infection [2, 3].

New endoscopic closure techniques are emerging and are thought to reduce the morbidity and postoperative complications. Recently, several authors have described the closure of TEF using an atrial stent, usually used for closure of a cardiac septal defect, with good results at 12 months of follow-up [6–8]. However, Buitrago et al. reported the death of a patient with massive hemoptysis after closure of a gastrobronchial fistula by an atrial stent [9]. This was due to progressive erosion of a branch of the bronchial artery by the self-expansible nature of the device. Sang et al. reported the use of a gastrointestinal stent specifically designed for TEF closure, made of laminated nitinol mesh with two self-expended disks [10]. The use of metal clips, biological glue, sclerosants, and electrocautery has also been reported in the literature, but the results are most often disappointing [3, 6–8]. To our knowledge, we report the first use of an endoscopic device made of porcine SIS for the closure of a TEF. This device is usually used for anorectal and rectovaginal fistula closure. This endoscopic technique is safe, with minimal intraoperative and postoperative complications. Convalescence is very short for patients with a rapid return to oral feeding (within one week postoperatively). However, this plug seems to be more appropriate in the management of long and narrow fistulas as its stability in large and short fistulas will probably be more difficult to obtain. We obtained complete closure of the fistula after 1 year of follow-up, allowing the patient to resume eating without difficulties.

## Conclusions

Tracheo-esophageal fistula is a difficult pathology to manage, and there are no established recommendations regarding therapeutic standards [6]. Its management must be based on accurate preoperative evaluation of the fistula and optimal preoperative preparation of the patient [2, 3]. Cervicothoracotomy is a major surgery and is potentially responsible for numerous postoperative complications [2, 4]. Treatment with endoscopic surgery is, when feasible, an alternative that reduces mortality with good results. We obtained, for the first time, a satisfactory result for TEF closure with the use of a porcine SIS plug, a natural extracellular matrix made of collagen and other non-collagenous components, that will help the healing of the tissue. Further studies are needed to confirm the efficacy of this new minimally invasive approach.

## Data Availability

The datasets supporting the conclusions of this article are available in the manuscript.

## Abbreviations

TEF: Tracheo-esophageal fistula
SIS: Small intestine submucosal
